# Intracranial spectral amplitude dynamics of perceptual suppression in fronto-insular, occipito-temporal, and primary visual cortex

**DOI:** 10.3389/fpsyg.2014.01545

**Published:** 2015-01-15

**Authors:** Juan R. Vidal, Marcela Perrone-Bertolotti, Philippe Kahane, Jean-Philippe Lachaux

**Affiliations:** ^1^INSERM U1028, CNRS UMR5292, Lyon Neuroscience Research Center, Brain Dynamics and Cognition Team, Lyon – Université Claude BernardLyon 1, Lyon, France; ^2^University Grenoble Alpes, LPNC, F -38040 GrenobleFrance; ^3^CNRS, LPNC, UMR 5105, F -38040 GrenobleFrance; ^4^CHU Grenoble and Department of Neurology, INSERM U704, F -38043 GrenobleFrance

**Keywords:** perceptual suppression, broadband gamma, conscious perception, anterior insula, primary visual cortex, visibility, intracranial EEG, contrast adaptation

## Abstract

If conscious perception requires global information integration across active distant brain networks, how does the loss of conscious perception affect neural processing in these distant networks? Pioneering studies on perceptual suppression (PS) described specific local neural network responses in primary visual cortex, thalamus and lateral prefrontal cortex of the macaque brain. Yet the neural effects of PS have rarely been studied with intracerebral recordings outside these cortices and simultaneously across distant brain areas. Here, we combined (1) a novel experimental paradigm in which we produced a similar perceptual disappearance and also re-appearance by using visual adaptation with transient contrast changes, with (2) electrophysiological observations from human intracranial electrodes sampling wide brain areas. We focused on broadband high-frequency (50–150 Hz, i.e., gamma) and low-frequency (8–24 Hz) neural activity amplitude modulations related to target visibility and invisibility. We report that low-frequency amplitude modulations reflected stimulus visibility in a larger ensemble of recording sites as compared to broadband gamma responses, across distinct brain regions including occipital, temporal and frontal cortices. Moreover, the dynamics of the broadband gamma response distinguished stimulus visibility from stimulus invisibility earlier in anterior insula and inferior frontal gyrus than in temporal regions, suggesting a possible role of fronto-insular cortices in top–down processing for conscious perception. Finally, we report that in primary visual cortex only low-frequency amplitude modulations correlated directly with perceptual status. Interestingly, in this sensory area broadband gamma was not modulated during PS but became positively modulated after 300 ms when stimuli were rendered visible again, suggesting that local networks could be ignited by top–down influences during conscious perception.

## INTRODUCTION

The flow of our conscious perceptual experiences evidently relate to the ongoing changes in our sensory inputs. In addition to establishing the limits between early sensory neural processing and subsequent conscious-related processing (Dehaene and Changeux, 2011) it has become a critical issue to scrutinize the conditions used in contrastive analyzes which typically compare brain activity for visible and invisible stimuli (Aru et al., 2012; de Graaf et al., 2012). For example, it might be relevant to *a priori* dissociate between perceptual invisibility that is caused by disabling sensory stimuli from reaching consciousness, which occurs when efficiently masking stimuli, from perceptual invisibility that is produced by suppressing a previously visible stimulus. Both invisibilities probably do not involve the same underlying neural mechanisms. In this study we focus on the latter which is called perceptual suppression (PS).

By definition, visual PS consists in making a visible object invisible despite ongoing retinal input. This has been achieved through flash suppression (Wolfe, 1984; Wilke et al., 2003, 2006; Tsuchiya and Koch, 2005). In binocular vision PS can also be induced by background motion, as in motion-induced blindness (MIB; Bonneh et al., 2001, 2014), by transient contrast decreases (May et al., 2003; Simons et al., 2006), or by presenting small visual transients throughout the visual field after short fixation (Kanai and Kamitani, 2003). Sudden contrast reductions have a strong impact so as to make complex visual scenes vanish from conscious perception for a prolonged period (Simons et al., 2006). The latter type of PS has been described and characterized at a psychophysical level, yet remains largely unexplored at the neural level, but could reveal important insights into the neural basis of conscious visual perception.

While previous studies explored the modulation of cortical spectral responses during PS (Wilke et al., 2006; Keliris et al., 2010; Panagiotaropoulos et al., 2012), we ignore if these responses are manipulation-specific, i.e., whether they depend on the type of experimental manipulation that induces PS, or whether they generalize to other types of manipulations inducing suppressions as well, such as those induced by contrast decreases, thereby reflecting general mechanisms underlying states of stimulus visibility and invisibility. To explore a novel experimental approach to conscious perception and to study neural activity modulation by PS we characterized the neural correlates of PS induced by stimulus contrast decrease (Simons et al., 2006), for the first time within human intracranial electroencephalographic (iEEG) recordings.

In our task participants passively fixated a typical Troxler ring-shaped stimulus while it underwent successive contrast increases and decreases, yet always remained physically on-screen. Within our experimental design, the same contrast level can be associated with either a visible or an invisible target. A behavioral account in our study showed that this effect elicited with specific parameters is perceptually robust and highly reproducible from trial-to-trial. We therefore implemented an adapted version of it for the subsequent intracranial recordings. In our analyzes we first focused on the global significant emergence of broadband gamma (50–150 Hz) and low-frequency alpha/beta (8–24 Hz) responses as compared to a baseline period. We estimated the modulation of these signals in sensory, limbic, and frontal cortices. This first analysis revealed which type of signal amplitude modulations, increases or decreases, are elicited by contrast changes (increases or decreases), respectively, linked to stimulus visibility (VIS) and invisibility (INV). However, this analysis does not directly link local network activity with stimulus visibility status processing, due to a possible confound with the sign of contrast change. Therefore, in a second analysis we pinpointed perceptual processing by contrasting the two previous conditions with a third condition, the stimulus offset (OFF), which also elicits stimulus disappearance but without stimulus sensory processing (see Materials and Methods). Summarizing, our paradigm uses perceptual invisibility during contrast decrease (INV), physical invisibility by removal of physical stimulus (OFF) and physical visibility by contrast increase (VIS).

Here we report that PS induced by contrast reduction elicits a decrease in low-frequency activity in distant cortical networks. Conversely, broadband gamma responses, especially in lateral occipital-temporal cortices, are positively modulated for this same physical manipulation which is associated with stimulus invisibility in our experimental design. Interestingly, when contrasting this condition with the two other conditions, respectively, stimulus visibility (by contrast increase) and stimulus invisibility (by stimulus offset), we found that in occipito-temporal cortices most effects were perceptual and were found in low-frequency amplitude modulations. In this same region, gamma-band activity modulation was divided between perceptual and sensory effects. Surprisingly, the temporal dynamics of the gamma-band amplitude modulation found in primary visual cortex (PVC, = V1), revealed unexpected patterns. There was no gamma-band response to stimulus contrast decrease (INV) and a slowly negative progressing gamma-band response to stimulus contrast increase (VIS). Interestingly, after 300 ms VIS responses displayed a positive and transient amplitude rebound, which could be signaling top–down feedback processing associated with the status transition from invisibility to visibility. Finally, we also contribute evidence on a role of frontal cortex in conscious perception by reporting that perceptual effects in the gamma-band appear earlier in fronto-insular networks than in occipito-temporal networks, conveying evidence supporting top–down signals in conscious perception that may arise from these networks. Globally, the results presented in this study shed light on a rich panel of underlying specific processes at work during PS with new insights for the neural basis and neural dynamics of conscious perception.

## MATERIALS AND METHODS

### STIMULUS AND TASK

#### Behavioral task

**FIGURE 1:**
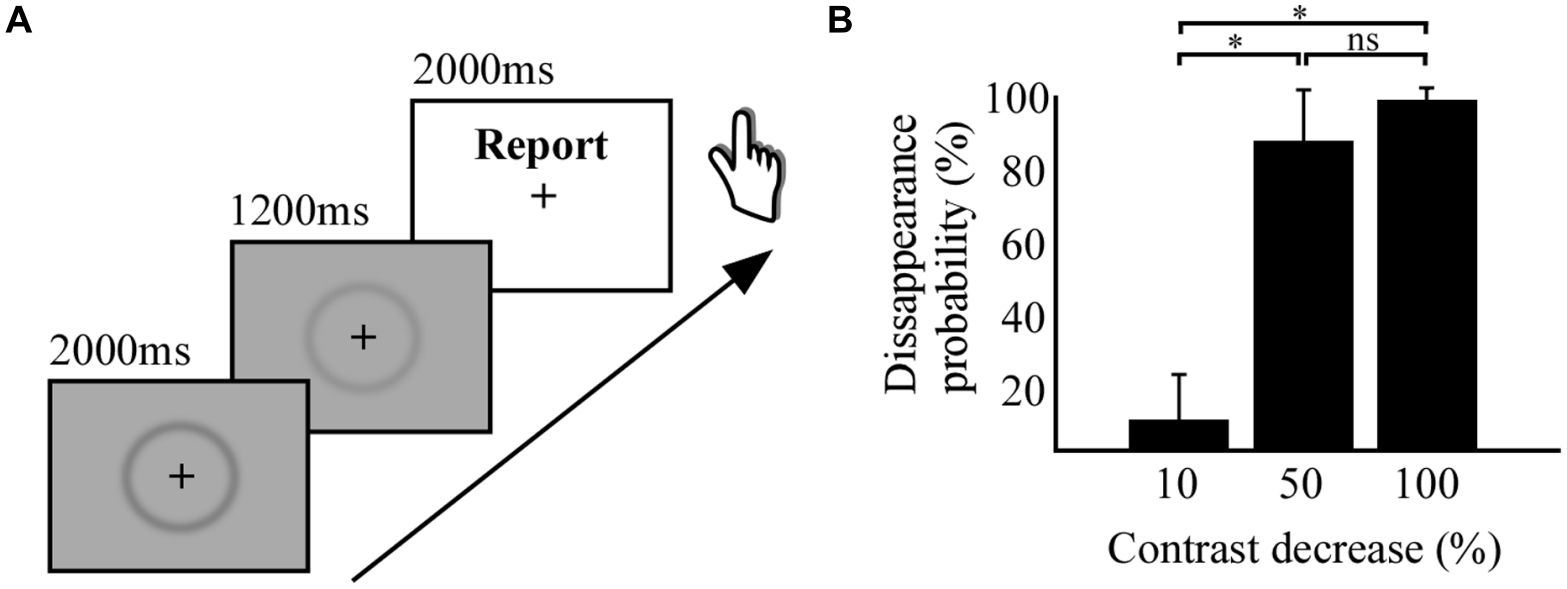
**Behavioral task and results. (A)** Experimental paradigm of perceptual suppression (PS) by stimulus contrast decrease. **(B)** Histogram displaying the results from the behavioral study, percentage of disappearance probability as a function of percentage of contrast decrease. * indicates significant difference between conditions.

The visual stimulus consisted of a centered dark gray fuzzy circle (Troxler stimulus, inner diameter: 10 cm; width 1 cm) on a gray background (**Figure 1A**). The image’s original contrast level was changed for three different stimuli with respective contrast decreases of 10, 50, and 100% (stimulus removal). A small black fixation cross was positioned at the center of the image and stimuli were presented on a screen positioned at ∼1 m from the participants. The behavioral task consisted in the following sequence of events (**Figure 1A**): after a 1–2 s of fixation the stimulus at 100% of contrast was presented for 2 s, followed by the presentation for 1.2 s of one of the three other stimuli with reduced. Thereafter, a white background screen was presented indicating three alternative response choices indicating full, partial, or null stimulus disappearance. Participants could respond during the 3 s duration of this period. The participants task was to rate on each trial his/her perceptual fading experience of the stimulus after the contrast change. Importantly, participants had the third option to indicate “partial disappearance” only when the stimulus did not fully disappear and remained partially visible. The disappearance probability of each stimulus was estimated by the ratio of the number of full disappearance reports divided by the total number of responses. As shown in **Figure 1B**, at 50% contrast decrease, the disappearance probability of the stimulus was near 95%, reflecting the strength of the PS effect. Similar effects have been shown in previous studies (May et al., 2003). The behavioral task was carried out on a laptop computer (screen: 60 Hz refresh rate).

#### Intracranial recording task

The same basic visual stimuli from the behavioral task was used in the experiment for the intracranial recordings. In this task, the stimulus contrast level indicated at 100%, which was equal to the 75% contrast level used in the behavioral task, was relatively reduced and increased to two different contrast levels: 50 and 150%. A small black fixation cross was positioned at the center of the circle and stimuli were presented on a screen positioned at ∼1 m from the participants. When increasing the contrast to its original level or higher the faded ring reappeared immediately. We used this PS effect in an original experimental design with two conditions, described in the following section.

**FIGURE 2:**
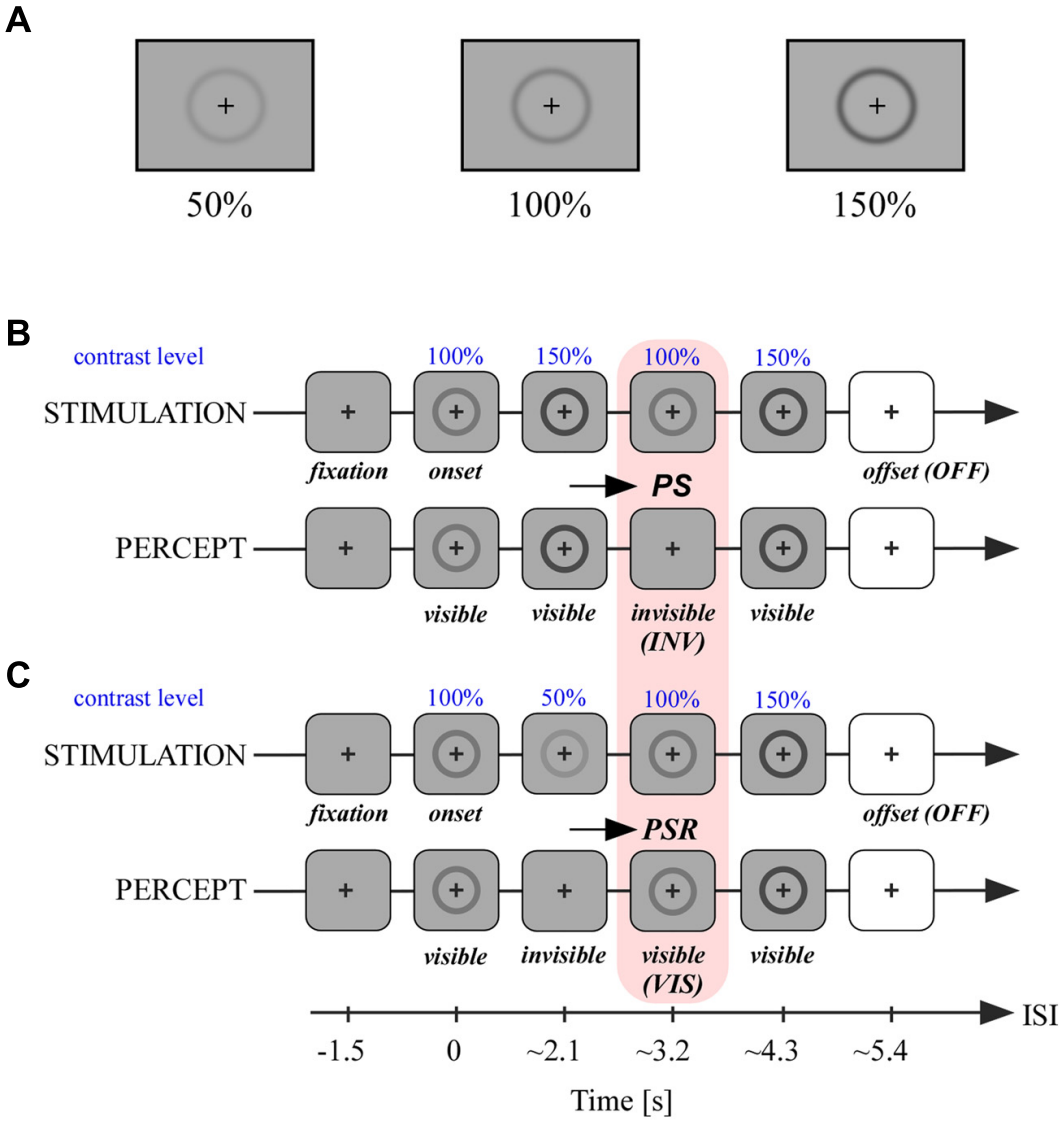
**Stimuli and experimental paradigm. (A)** Stimuli at different contrast levels as used in the experimental paradigm detailed in **(B,C)**. **(B)** In the first condition a ring-shaped stimulus at 100% contrast centered on a fixation cross was presented for ∼2.1 s. The following contrast levels of the stimulus were changed thereafter every ∼1 s in the following order: 150, 100, and 150%. When passing contrast level from 150 to 100%, PS occurred and the stimulus became invisible. After the last contrast change to 150% the stimulus was set off with a white screen. **(C)** The second condition was similar to **(A)** with a different pattern of contrast changes after stimulus onset: 50, 100, and 150%. When the stimulus contrast level passed from 100 to 50%, the stimulus became invisible; when thereafter the stimulus’s contrast increased again to 100%, the stimulus became visible again, a process called perceptual suppression release (PSR). We analyzed neural activity in conditions **(A)** and **(B)** within this second period where stimuli are both at 100% of contrast level but are either invisible **(A)** or visible **(B)**.

A trial consisted in four successive contrast changes of the same ring-shaped stimulus (**Figures 2B,C**). Each trial started with the presentation of a central fixation cross for a duration of 1.5 s, followed by the onset of the stimulus at 100% contrast for 2.2 s. In the first condition termed INV the contrast changes occurred in the following order and lasted 1.1 s: 150, 100, and 150%. The latency of stimulus onset or stimulus change that includes a temporal jitter of 0–100 ms is preceded by the ∼ symbol. The contrast variations produced stimulus invisibility in the third step when the stimulus contrast was decreased 50% (**Figure 2B**). In the second condition termed VIS the contrast changes occurred in the following order: 50, 100, and 150%. In this series the stimulus became invisible in the second step and was visible again in the third step when the contrast was increased 50%. Importantly, through these different contrast manipulations we could achieve for the same stimulus physical contrast level (100%) two different percepts, either an invisible or a visible stimulus, as illustrated by the pink box relating panels B and C in **Figure 2**. After the fourth step which increased the stimulus contrast from 100 to 150%, the stimulus was set off during the presentation of a white screen (offset, OFF). In 50% of trials the stimulus decreased 50% contrast for a brief 0.2 s before full stimulus offset. We used the other 50% of all trials’ offset (both conditions), who did not include this brief transient, in subsequent analyzes detailed in the data analysis section. Participants were instructed to fixate the central cross during all contrast changes and detect this very short stimulus disappearance before stimulus offset (white screen). This detection task was not related to prior perceived contrast changes and its only purpose was to keep participants focused throughout trials. This experimental design has the advantage to reduce neural processing related to the preparation of subjective report in the period of interest which involves mainly passive fixation of successive stimulus contrast and visibility changes (pink box, **Figures 2B,C**). After a short task training period which concerned the maintenance of gaze fixation throughout contrast changes participants reported a vivid experience that the stimulus disappeared (PS) and subsequently reappeared after contrast increase (PSR; **Figures 2B,C**). There were between 60 and 120 trials per condition, depending on the number of blocks (=30 trials) completed by each participant. The intracranial task was carried out on a desktop computer (screen: 60 Hz refresh rate).

#### Participants and intracranial recordings

The behavioral task included 10 healthy participants (five males, five females, mean age 32.8 ± 2.0 SEM), all had normal or corrected-to-normal vision and provided written informed consent. Intracranial recordings were obtained from 9 neurosurgical patients (five females, four males, mean age: 28.7 ± 3.3 SEM) with intractable epilepsy at the Epilepsy Department of the Grenoble University Hospital and at the Epilepsy Department of the Neurological Hospital in Lyon. All participants had normal or corrected-to-normal vision, gave written informed consent, and the experimental procedures were approved by the Institutional Review Board and by the National French Science Ethical Committee. Electrode implantation was performed according to regular procedures and all target structures for the presurgical evaluation were selected strictly according to clinical considerations with no reference to the current study. Eleven to fifteen semi-rigid, multi-lead electrodes were stereotactically implanted in each patient, with 10–15 recording sites on each electrode (2 mm wide, 3.5 mm center-to-center; Jerbi et al., 2009b; Lachaux et al., 2012). Intracranial EEG recordings (iEEG) were conducted using a video-iEEG monitoring system (Micromed), which allowed the simultaneous data recording from 128 depth-EEG electrode sites. All electrode contacts were identified on a postimplantation MRI showing the electrodes, and coregistered on a pre-implantation MRI. MNI and Talairach coordinates were computed using the SPM (http://www.fil.ion.ucl.ac.uk/spm/) toolbox.

#### Data analysis

**FIGURE 3:**
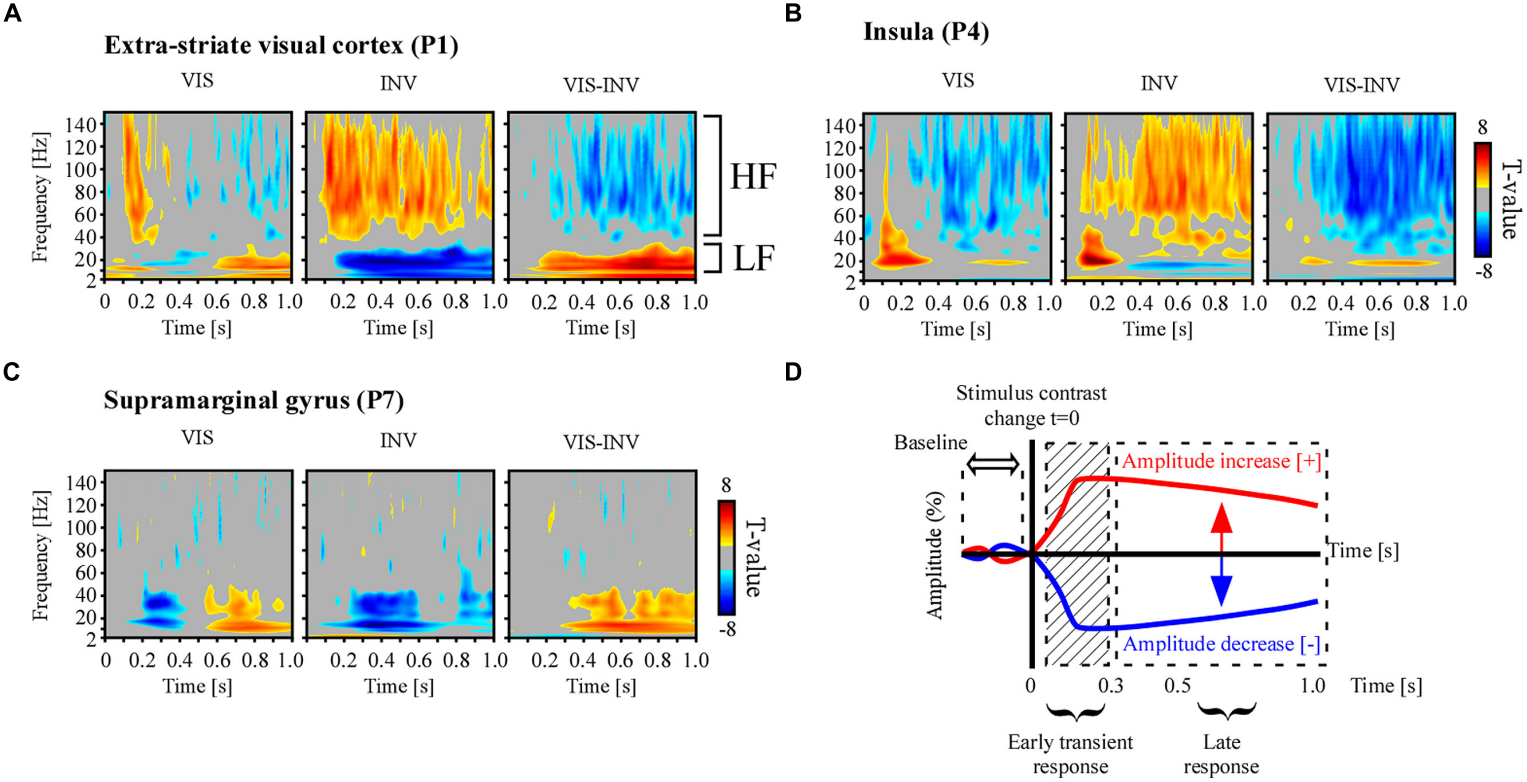
**Illustrative examples of individual spectral responses and neural emergence testing**. Time-frequency statistical maps of neural response emergence in PS, PSR and the comparison between the two conditions in **(A)** extra-striate visual area (MNI coordinate: [-39 -75 2]), **(B)** anterior insular cortex (MNI coordinate: [-37 18 9]) and **(C)** supramarginal gyrus (MNI coordinates: [58-21 18]). Broadband gamma power increase and low-frequency power decrease, as compared to pre-stimulus baseline, were observed simultaneously in various recording sites during PS, though in some sites only one of two effects were present, as exemplified in **(C)**. The opposite effect pattern was observed for PSR. All TF T-maps are scaled between -8 and 8. **(D)** To study spectral emergence, i.e., the orientation of the different spectral responses as compared to the average baseline level, we averaged the amplitude in the late part of the neural response, bypassing the initial transient response induced by the changing sensory input.

Data were sampled at 512 Hz and each recording site was referenced to its adjacent neighbor, (bipolar montage). In this study we extracted spectral responses from neural signals using two spectral analysis techniques: wavelets and Hilbert transform. Based on prior knowledge from previous studies involving visual induced spectral responses in intracranial recordings (Vidal et al., 2010, 2011; Dalal et al., 2011; Hamame et al., 2014; Perrone-Bertolotti et al., 2014) we targeted two frequency bands of interest: the broad gamma band (50–150 Hz) and the low-frequency band (8–24 Hz). As proposed elsewhere (Lachaux et al., 2012), the broad gamma band is currently considered as a functional correlate of local cortical activation while the low frequency bands might reflect long-range interactions involved in top–down processing (Siegel et al., 2012). The amplitude responses within these bands were extracted with a Hilbert transform that is explained further in the text. However, in order to verify that such prior based frequency band selection indeed contained effective signal modulation we first computed a standard time-frequency (TF) wavelet decomposition within the 2–150 Hz band. To estimate the significance of these post-stimulus responses within this frequency range, as compared to baseline ([–200 to –50]ms) power in a total time window from -200 to 800 ms post-stimulus, we used a paired *t*-test across trials at each recording sites. Illustrative examples of this analysis are shown in **Figure 3**.

To increase signal-to-noise ratio of spectral neural signals in the low and high frequency bands we thus opted to compute the amplitude envelope in the averaged high and low frequency intervals, respectively. We therefore computed the Hilbert transform to evaluate the significance of these neural responses with a lower degree of complexity across all electrodes. To obtain high-frequency activity amplitudes, for example between 50 and 150 Hz, we applied the following processing steps. First we bandpass filtered iEEG signals in multiple successive 10 Hz-wide frequency bands (e.g., 8–10 bands as [50–60 Hz], [60–70 Hz], etc.) using a zero phase shift non causal finite impulse filter with 0.5 Hz roll-off. For the low-frequency interval between 8 and 24 Hz we used 4 Hz-wide bands. Next, for each bandpass filtered signal we computed the envelope using a standard Hilbert Transform. For each frequency bin (ex: [60–70 Hz]) the time-varying amplitude was divided by its mean across the entire recording period of the experiment and multiplied by 100. This yields instantaneous amplitude envelope values as percentage-of-the-mean. Finally, the envelope signals computed for each consecutive band were averaged to provide one single time series across the entire recording session. The obtained envelopes had a sampling rate of 64 Hz. This procedure has been previously used in various studies in our group (Jerbi et al., 2010; Juphard et al., 2011; Ossandon et al., 2012; Vidal et al., 2012). To estimate the post-stimulus neural activity modulations vs. baseline (neural emergence), excluding the initial activity transient produced by neural sensory changes, we estimated the difference between the average amplitude in the 300–800 ms post-stimulus time interval with the averaged pre-stimulus (from -200 to -50 ms) baseline amplitude response by the means of a paired *t*-test, across all electrodes and corrected for multiple comparisons across this dimension with a false discovery rate test (FDR; Genovese et al., 2002). To estimate the difference in neural response amplitude between experimental conditions (visible, invisible and offset) averaged across time, we used a repeated measures analysis of variance (ANOVA) combined with a *post hoc* Tukey–Kramer test. All statistical *p*-values were FDR-corrected (False Discovery Rate, Genovese et al., 2002) for multiple comparisons across the number of electrodes. When these analyzes were performed on time resolved data, we equally corrected for multiple comparisons across time samples per ROI. We additionally considered effects significant and of interest when repeated on at least five consecutive time samples. All analyzes were performed using Matlab (The Mathworks, Inc., Natick, MA, USA).

In this study we focused mainly on two frequency ranges. We analyzed a low and high-frequency interval according to preliminary TF analysis (see Results section). The low-frequency interval ranged from 8 to 24 Hz including mainly alpha and beta band frequency response intervals. The high gamma band interval ranged from 50 to 150 Hz. This interval captures the broadband spectral responses that appear commonly in human intracranial recordings (Ossandon et al., 2012; Vidal et al., 2014). Note that although iEEG signals provide access to neuronal population activity across a wide range of frequencies especially in the gamma band, we focused here on broadband HFA because it has been closely related to population-level neuronal spiking activity (Manning et al., 2009; Ray and Maunsell, 2011) and it is increasingly used as a proxy for active cortical processing (Buzsaki and Wang, 2012; Lachaux et al., 2012). In line with human EEG studies on the link between low and high frequency bands and BOLD signal (Goldman et al., 2002; Laufs et al., 2003; Scheeringa et al., 2011) the high gamma band amplitude fluctuation, also appears to correlate with the fMRI BOLD signal (Logothetis et al., 2001; Lachaux et al., 2007a; Worrell et al., 2012) and has been shown to help neural decoding and real-time mapping of cognitive brain function and processes (Lachaux et al., 2007b; Jerbi et al., 2009a; Hamame et al., 2012).

## Results

To use PS by stimulus contrast decrease for subsequent intracranial recordings we first investigated the strength of the PS by the level of contrast decrease in a group of healthy participants. The experiment revealed that small contrast decreases of -10% has only a marginal effect on disappearance probability (8% disappearance probability), as compared to the medium contrast decrease of -50%, which induced 89% of disappearance probability (**Figure 1B**). A repeated measures ANOVA revealed a significant difference [*F_(29)_* = 178.1; *p* < 0.001) between the disappearance probability of the three levels of contrast decreases (-10, -50, and -100%) and the *post hoc* Tuckey–Kramer test revealed that the -10% condition was different from the other two conditions. We concluded from this first study that decreasing half the contrast could achieve full PS of the visual stimulus, statistically indissociable from full stimulus removal (-100% contrast).

We recorded from a total of 976 electrodes widely distributed throughout the cortex (nine patients). The experimental paradigm is detailed in **Figures 2B,C**. We focused our analyzes on the time-window indicated by the pink box (**Figures 2B,C**) to compare neural activity elicited by stimulus visibility (VIS) and invisibility (INV) at identical physical stimulus contrast (**Figures 2B,C**). Based on evidence from previous studies on spectral responses of visual processing in intracranial recordings (Vidal et al., 2010) we expected strong modulations within the broad high-gamma band (50–150 Hz) and a low-frequency interval grouping both alpha and beta frequency ranges (8–24 Hz). These signals were extracted with a Hilbert transform procedure (see Materials and Methods section) and subsequently statistically analyzed (detailed in the subsequent paragraph). From these results we chose three representative electrodes in three different cortical areas to proceed to a full TF decomposition with a wavelet procedure, and show the described effects can also be visible within the full TF interval. The results of this analysis are described in the next paragraph.

Time-frequency statistical analyzes revealed a significant post-stimulus spectral power modulation relative to pre-stimulus-change baseline level (-200 to -50 ms). **Figures 3A–C** show statistically significant spectral power changes for the two experimental conditions, visible (VIS) and invisible (INV), obtained from three illustrative electrodes from three different participants, and the comparison statistics between the two conditions. First, stimulus invisibility induced a sustained power increase in a rather broad portion of the TF spectra (50–150 Hz). This increase occurred beyond the initial transient activity increase yielded by the contrast change. Simultaneously it induced a sustained power decrease in low-frequency bands, including alpha (8–12 Hz) and beta band (16–24 Hz; Middle panels in **Figures 3A,B**). In the right panel of **Figure 3A** the two distinct frequency intervals are marked as HF, high–frequency, and LF, low-frequency. An opposite response pattern was observed for stimulus visibility obtained during PSR, with a broadband gamma power suppression as the contrast increased (Left panels in **Figures 3A,B**). In the low-frequency range (8–24 Hz) power modulations occurred in opposite sign as for the respective high-frequency modulations. Stimulus contrast increases often elicited power increases in the low-frequency interval while stimulus contrast decreases induced power decrease in this band (**Figures 3A–C**). Though power modulations in both frequency intervals occurred in a variety of brain areas (occipital, temporal, frontal, limbic, and parietal), they did not systematically occur simultaneously at the same sites, as illustrated in **Figure 3C**, for an electrode located in the supramarginal gyrus.

**FIGURE 4:**
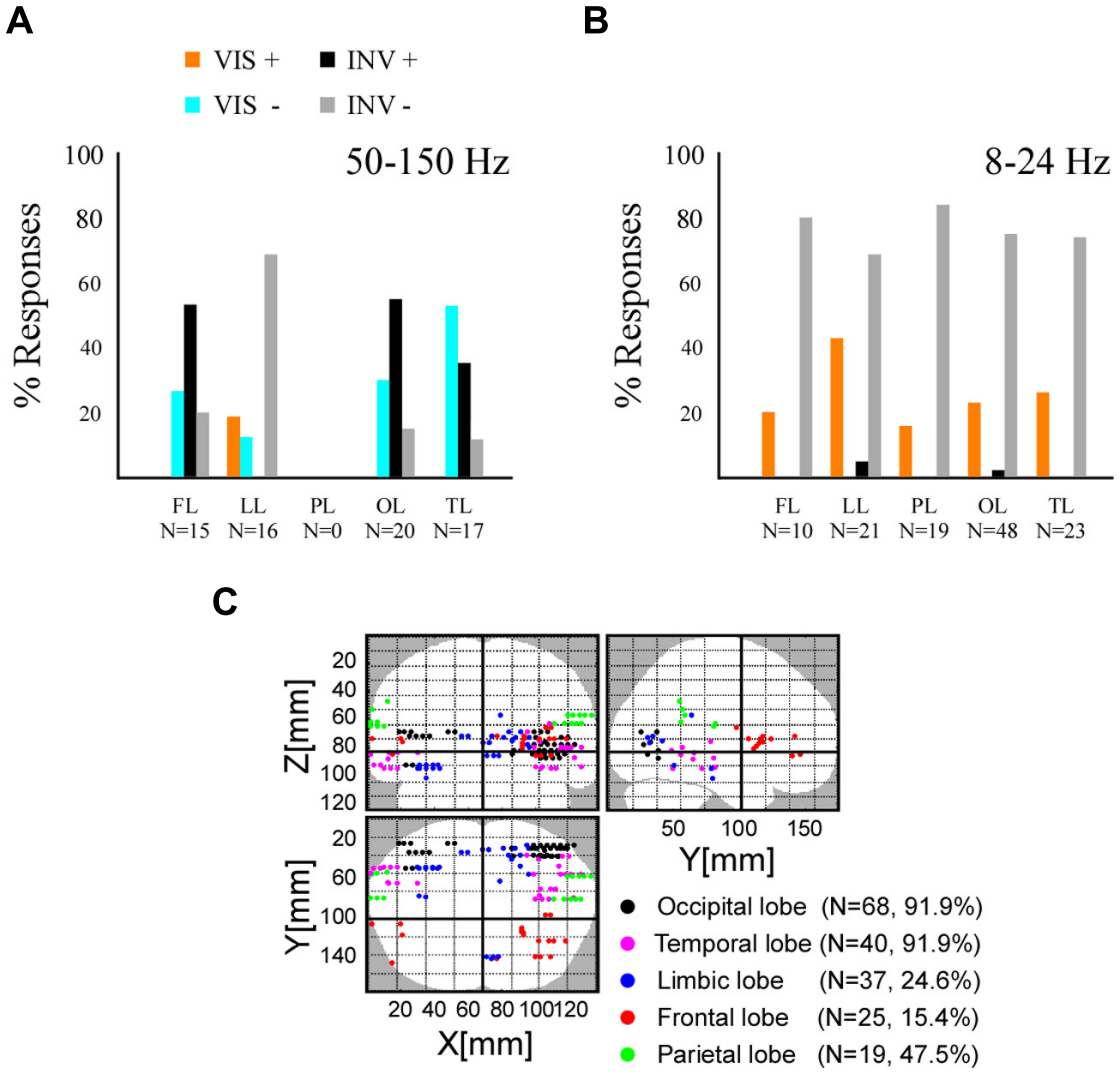
**Spectral emergence for high and low frequency responses across five cerebral lobes. (A)** Histogram displaying the percentage of the broadband gamma (50–150 Hz) activity emergence in frontal lobe (FL), limbic lobe (LL), parietal lobe (PL), occipital lobe (OL), and temporal lobe (TL). Stimulus visibility, VIS, and related activity increases (+) and decreases (-). Stimulus invisibility, INVIS, and related activity increases (+) and decreases (-). **(B)** Histogram displaying the percentage of the low-frequency activity (8–24 Hz) emergence, same conventions as in **(A)**. **(C)** Brain wide distribution of recording sites displaying either type of neural emergence response.

Because contrast changes induced either sustained stimulus visibility or invisibility, beyond the contrast transition, we next focused our analysis on the interval after the initial transient neural response induced by the change in contrast, which occurs in the first couple of 100 ms. We therefore excluded the amplitude signals between 0 and 300 ms post-stimulus change (**Figure 3D**) and operated all subsequent statistical comparisons on the average signal amplitude in the 300–800 ms post-stimulus time window. Though some authors consider conscious processing to initiate in higher visual cortices after the sensory-gated feedforward sweep, i.e., around 100 ms (Lamme, 2003, 2006), others consider this to occur later in time and involving feedback processing from frontal areas, around 300 ms after stimulus onset (Dehaene et al., 2006). After this temporal limit in our recordings, stimulus invisibility by contrast decreases elicited gamma-band amplitude increase mostly in occipital, temporal and frontal cortex (**Figure 4A**). In a majority of medial cortices in temporal and frontal regions (i.e., limbic lobe), gamma-band activity decreased for invisible stimuli. Stimulus visibility elicited fewer responses, and showed mostly gamma-band decreases in temporal, occipital and frontal cortex (**Figure 4A**). Stimulus invisibility induced low-frequency amplitudes suppression on most recording sites across frontal, limbic, parietal, occipital, and temporal lobes (**Figure 4B**). Stimulus visibility induced mostly amplitude increases in all responsive cortical networks (**Figure 4B**). The electrode locations eliciting all the above significant effects within the different cortical lobes are depicted in **Figure 4C**. Importantly, though these effects occur, respectively, for periods of stimulus visibility and invisibility, they also correspond to opposite steps in contrast change (respectively, contrast increase and contrast decrease) and their singular contrastive analysis may not yield per se neural correlates of conscious perception (Aru et al., 2012; de Graaf et al., 2012). Moreover, they may also be affected by the previous states and state transitions, which also imply different contrast levels and contrast level transitions. See the discussion section for further implications of this particularity of our experimental design.

In order to further discriminate the functional processing that might be reflected by local networks we further characterized PS by comparing the amplitude responses elicited by contrast reduction (to 50%) to those elicited by full stimulus offset (**Figures 2B,C**), in line with previous studies and to dissociate sensory from perceptual processing components of neural responses (Wilke et al., 2006, 2009). Although the offset condition differ in terms of low-level physical properties such as time of presentation and background color, we used this contrast mainly because it elicits a phenomenal perceptual experience of stimulus disappearance highly similar to the one produced by the condition of stimulus contrast reduction. We especially considered neuronal populations located in occipito-temporal and frontal cortical regions who process rather highly integrated visual information and whose activity is modulated by conceptual content of images and their conscious perception (Kreiman et al., 2000, 2002; Quiroga et al., 2007, 2008a,b; Vidal et al., 2010, 2014; Libedinsky and Livingstone, 2011; Panagiotaropoulos et al., 2012). To avoid any interference from the detection task at the end of the contrast-change series, we only used those trials (50%) which were not associated with a behavioral response (see Materials and Methods) but who were equally distributed among the VIS and INV conditions.

**FIGURE 5:**
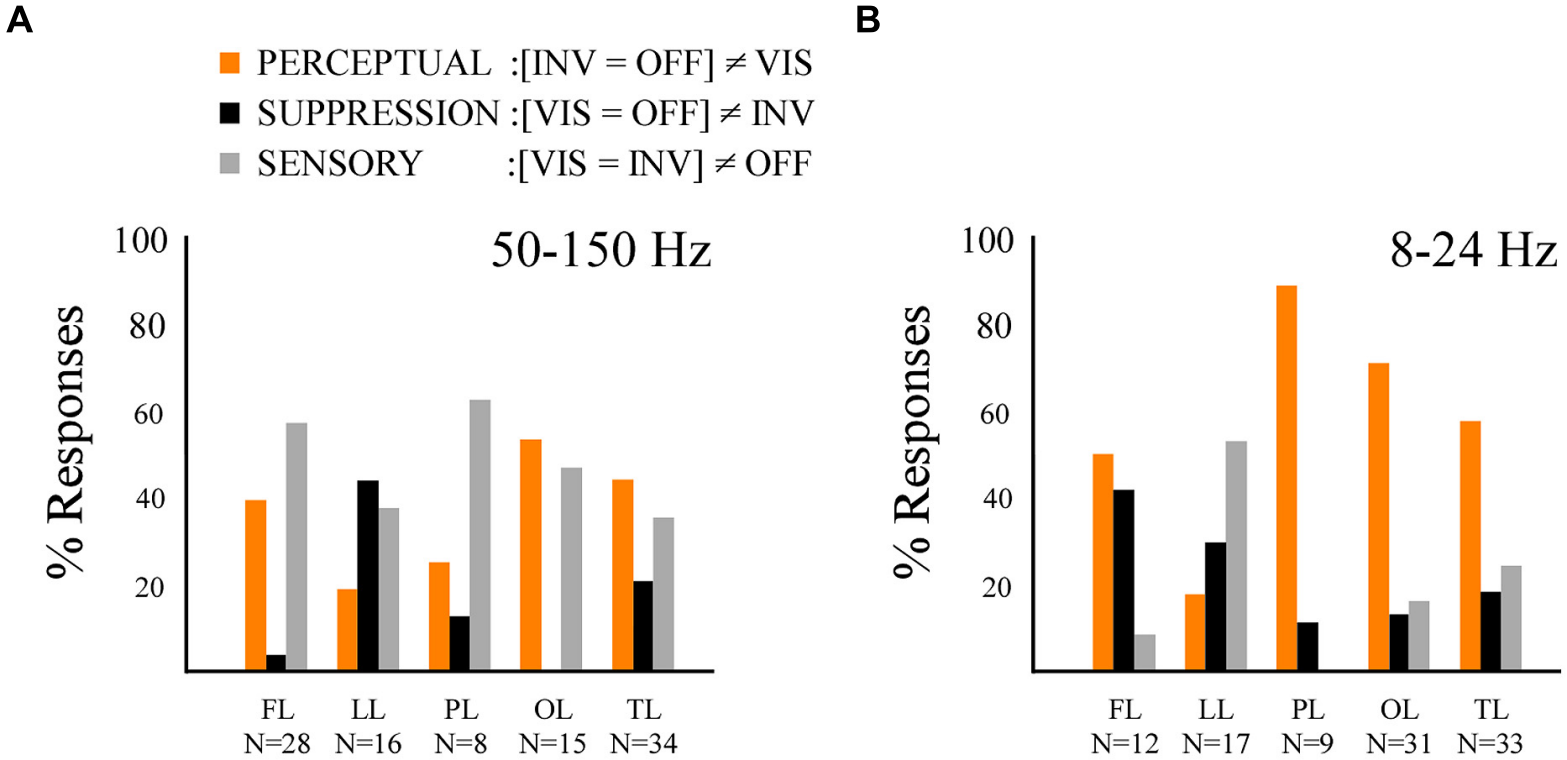
**Results from the multiple comparison test revealing the perceptual, suppression and sensory effects. (A)** Histogram displaying the percentage of response per effect type within the broad gamma band (50–150 Hz) across the five lobes, same conventions as in **Figure 4A**. **(B)** Histogram displaying the percentage of effect type response within the low-frequency band (8–24 Hz) across the five lobes, same conventions as in **Figure 4A**.

By contrasting these three conditions, instead of two conditions, we were able to obtain more information on the involvement of the local neural network in three types of functional processing: (a) *perceptual processing (PERCEPTUAL)*, which differentiates conditions regarding stimulus visibility, independently of stimulus presence or absence, (b) *sensory processing (SENSORY)*, which differentiates conditions of stimulus processing independently of stimulus visibility or not, and (c) *suppression processing (SUPPRESSION)*, which differentiates the conditions eliciting stimulus invisibility while processing a sensory stimulus, from the two other conditions. Importantly, these effects are considered independently of sign. We compared the time-averaged amplitude of the three conditions (one way repeated measures ANOVA and Tuckey–Kramer *post hoc* test, FDR-corrected for multiple comparisons), respectively, for low-frequency and high-frequency responses, and grouped the effects between these conditions into three previously defined functional categories: PERCEPTUAL effects ([INV = OFF]≠VIS), SENSORY effects ([VIS = INV]≠OFF) and SUPPRESSION effects ([VIS = OFF]≠INV). In the high-frequency band we found all three response types in most cortical areas (**Figure 5A**). PERCEPTUAL effects ([INV = OFF]≠VIS) in the high-frequency range were found in frontal, temporal, and occipital cortices (**Figure 5A**). SENSORY effects ([VIS = INV]≠OFF) were effectively represented in occipital, parietal and temporal cortex, and more surprisingly were also found in frontal sites (**Figure 5A**). Interestingly, SUPPRESSION effects ([VIS = OFF]≠INV) were mostly absent in occipital cortex and mainly observed in medial and lateral cortices of the temporal lobe (**Figure 5A**). Low-frequency responses displayed proportionally overall more PERCEPTUAL effects ([INV = OFF]≠VIS) in occipital, temporal and parietal networks (**Figure 5B**). SUPPRESSION effects ([VIS = OFF]≠INV) were mostly observed in frontal and limbic cortices, while SENSORY effects ([VIS = INV]≠OFF) were mostly present in the frontal and limbic lobe.

**FIGURE 6:**
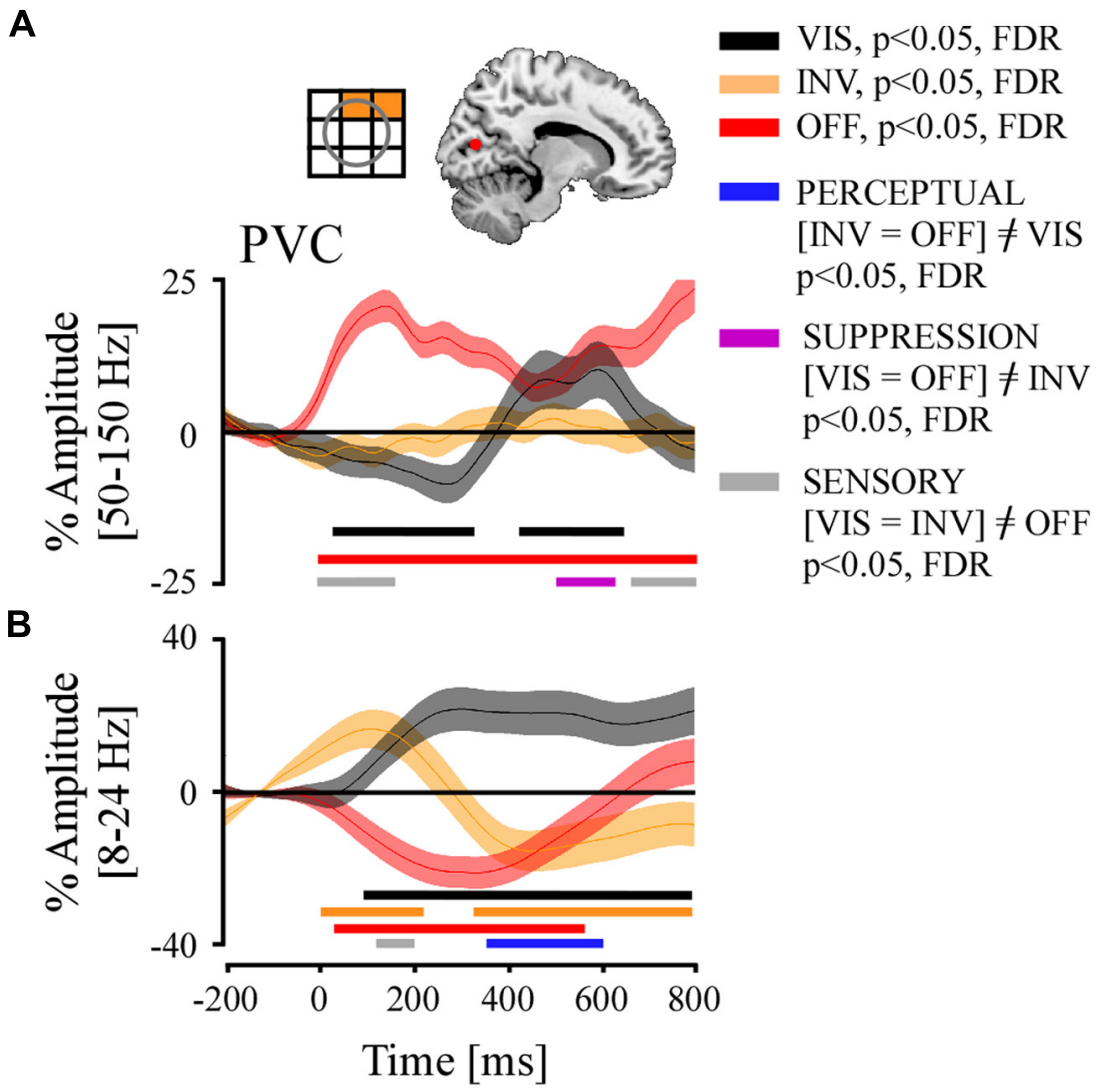
**Grand average of time-resolved high and low frequency responses and their comparison statistics for stimulus visibility (VIS), invisibility (INV), and stimulus offset (OFF) in the PVC**. Above **(A)**, left panel, a retinotopic receptive field map of recording sites, and right panel, localization of electrodes projected on MNI reference brain. **(A)** Broadband gamma responses, significant time-samples of neural emergence test and of condition contrasts. **(B)** Low-frequency responses, same conventions as in **(A)**.

We now focus on the PVC. In this study one participant (P4) had three electrodes implanted in PVC which have been retinotopically mapped to mid- and upper right quadrant of the visual field (see **Figure 6** for a representation). We were specifically interested in the frequency-specific activity modulation across trials, considering previous studies on PS who studied spike rate and gamma-band activity modulation in V1 of the macaque brain. We found various interesting results (**Figure 6**):

(1) Stimulus offset (OFF) positively modulated broadband gamma activity during the whole temporal interval. The effect started very early due of the temporal smearing inherent to the spectral extraction method (Hilbert) used in our analysis. Interestingly, there was a total absence of amplitude modulation in the INV condition, which involved a stimulus contrast decrease entailing stimulus invisibility. In the VIS condition where the stimulus’s contrast was increased, we first observed a slow progressive negative amplitude modulation, which appeared unrelated to the contrast change of the stimulus but did relate to the perceptual state in the previous period (**Figure 6**). 312 ms after contrast increase we observed a positive amplitude re-bound that shortly after 400 ms became positively modulated as compared to baseline, and lasted until 670 ms.
(2) The repeated measures ANOVA of the three conditions (VIS, INV, and OFF) across all trials and for each time bin from the three recording sites in PVC showed a very early SENSORY effect ([VIS = INV]≠OFF). A SUPPRESSION effect ([VIS = OFF]≠INV) appeared again between 500 and 630 ms, which again was entailed by a SENSORY effect. No significant PERCEPTUAL ([INV = OFF]≠VIS) effects were found (*p* > 0.05).
(3) Low frequency activity after stimulus offset (OFF) showed an immediate significant power decrease until 550 ms after which it re-bounded positively. The INV condition appeared initially to be under the influence of the prior state, but after 300 ms became and remained negatively modulated until the end of the time period. The VIS condition induced an early and sustained significant positive amplitude modulation until the end of the analyzed time period.
(4) The comparison between conditions revealed first an early <200 ms SENSORY effect ([VIS = INV]≠OFF). From 350 to 600 ms we observed a PERCEPTUAL effect ([INV = OFF]≠VIS).

Finally, we explored the dynamics of the PERCEPTUAL effects across different frontal and temporal recordings sites. It has been shown that neuronal discharges in temporal cortex (Sheinberg and Logothetis, 1997) and in lateral prefrontal cortex (Panagiotaropoulos et al., 2012) reflect unambiguously stimulus visibility at similar latencies, yet no study on PS has ever directly compared these latencies simultaneously in intracranial recordings to explore the information flow of perceptual information between these structures. Two participants within the group of patients who took part of our study had electrodes implanted in both regions (P4 and P7). Though we did not have access to multi-unit or single-unit recordings in those patients, we analyzed broadband gamma amplitude modulations which are often considered as a proxy of spiking activity at the population level (Nir et al., 2008; Manning et al., 2009; Ray and Maunsell, 2011; Buzsaki and Wang, 2012).

**FIGURE 7:**
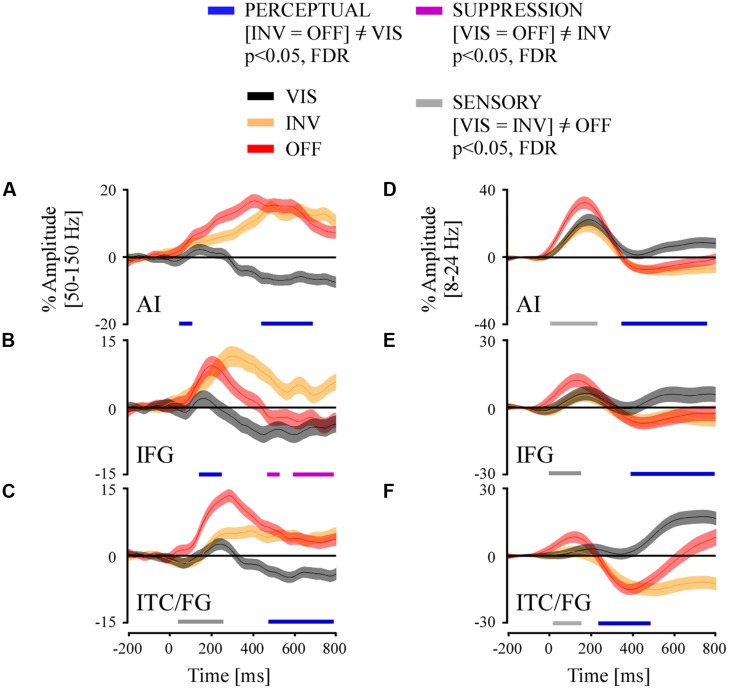
**Grand average of time-resolved high and low frequency responses and their comparison statistics for stimulus visibility (VIS), invisibility (INV), and stimulus offset (OFF) in three ROI: (A,D) anterior insula (AI), **(B,E)** inferior frontal gyrus and **(C,F)** inferior temporal cortex/fusiform gyrus (ITC/FG)**. The comparison statistics revealed three effect types, PERCEPTUAL (blue) and SUPPRESSION (purple) and SENSORY (gray), for broadband gamma signals **(A–C)** and low-frequency amplitudes **(D–F)**.

Twenty-four recording sites from participants elicited PERCEPTUAL effects in the broadband gamma range in three regions of interest: temporal cortex (fusiform gyrus/inferior temporal cortex, ITC/FG, *N* = 11), anterior insula (AI, *N* = 7) and inferior frontal gyrus (IFG, *N* = 7). To estimate the latency of these average PERCEPTUAL effects ([INV = OFF] ≠VIS) we applied a repeated measure ANOVA (conditions: VIS, INV, and OFF) across all trials per region of interest across the full time-interval 0–800 ms, and corrected for multiple comparisons in time. We considered the first significant time sample of a minimum of five consecutive time sample. Here we report that PERCEPTUAL effects initiated, respectively, in the AI at 65 ms [*F_(1992)_* = 5.16; *p* < 0.01; *post hoc* T–K test, *p* < 0.05], in the IFG at 174 ms [*F_(1735)_* = 8.44; *p* < 0.0005; *post hoc* T–K test, *p* < 0.05] and in the ITC/FG at 483 ms [*F_(2978)_* = 65.3; *p* < 0.0005; *post hoc* T–K test, *p* < 0.05] (**Figures 7A–C**). In all three ROI, the INV condition elicited a positive amplitude modulation while the VIS condition elicited a negative modulation (**Figures 7A–C**). We conducted the same analysis for the low-frequency responses at these three ROI. We found that PERCEPTUAL effects initiated, respectively, in the ITC/FG at 268 ms [*F_(3007)_* = 6; *p* < 0.05; *post hoc* T–K test, *p* < 0.05], in AI at 377 ms [*F_(2022)_* = 6.2; *p* < 0.05; *post hoc* T–K test, *p* < 0.05] and in the IFG at 424 ms [*F_(1786)_* = 5.15; *p* < 0.05; *post hoc* T–K test, *p* < 0.05] (**Figures 7D–F**).

## Discussion

Currently the study of conscious visual perception can rely on a wide variety of experimental paradigms (Kim and Blake, 2005; Koch and Tsuchiya, 2007; Dehaene and Changeux, 2011), some of these produce PS which is described as the subjective experience of seeing visible items become invisible. In this study we focused on the neural correlates of conscious visual perception, using an experimental design which produced PS through the transient contrast decrease of a single and entire stimulus (May et al., 2003; Simons et al., 2006). This effect resembles the visual fading effect described by Troxler (1804) yet can be manipulated at wish after a short visual adaptation. By manipulating the temporal order of contrast decreases and increases we obtained in a time period of interest two conditions with equal and unchanging stimulus contrast which elicited two different perceptual states, stimulus visibility, and stimulus invisibility. We recorded human intracranial electrophysiological activity in nine patients and analyzed spectral responses in the broadband gamma 50–150 Hz range and in the low-frequency alpha/beta 8–24 Hz range. In a first phase we analyzed globally these signals across recording sites in cortical ROI. We report that stimulus invisibility by PS produces a sustained energy decrease in low-frequency bands across recording sites in many regions including occipital, temporal, limbic, parietal, and frontal cortex, while stimulus visibility achieved through suppression release produced a somewhat less strong opposite effect of energy increase. We also report a majority of broadband gamma activity increases during PS in occipital, temporal, and frontal cortices. Overall, stimulus invisibility through PS elicited neural responses across a larger number of recording sites than stimulus visibility. Moreover, by comparing these conditions to a shortly later stimulus offset we were able to describe signal effects as SENSORY, PERCEPTUAL, or SUPPRESSION related. We also report that PERCEPTUAL processing within the low-frequency band was largely present in occipital, temporal, parietal, and frontal cortices. Finally, in a second analysis phase we focused on time-resolved neural activity modulation to estimate the latencies of PERCEPTUAL effects in three ROI: AI, IFG, and ITC/FG. We were also able to analyze neural activity from PVC in one participant. Before discussing our results in the light of previous findings and theoretical accounts, we outline the main differences of our experimental approach as compared to previous PS paradigms, in order to understand some discrepancies between our results and those previously reported.

### STIMULUS INVISIBILITY THROUGH TRANSIENT CONTRAST DECREASE

Experimental paradigms that induce the perceptual disappearance of a stimulus such as MIB (Bonneh et al., 2001) and flash suppression (Wilke et al., 2003; Tsuchiya and Koch, 2005) have in common that stimulus invisibility is produced by the presentation of a competing stimulus in the same visual field. These sensory manipulations introduce competitive interactions between different stimuli in the visual field or between sensory streams thereby biasing sensory selection for further conscious processing (and perception) of the newly presented or changing stimulus at the expense of the constant and static ones. Stimulus invisibility could thus be related to the parallel processing of the novel stimulus that becomes visible and whose sensory processing could generate competing neuronal interactions for selective attention. In our PS’s experimental approach stimulus invisibility was achieved without the processing of a novel visible stimulus, avoiding competing interactions between distinct sensory representations for sensory or attentional selection, which are known to modulate neural processing within early visual cortices and beyond in temporal networks (Moran and Desimone, 1985; Desimone and Duncan, 1995; Reynolds and Desimone, 1999; Kastner and Ungerleider, 2000; Reynolds et al., 2000; Fries et al., 2001, 2008; Bichot et al., 2005). However, at a local neural networks level, especially in sensory visual cortices, competitive interactions could occur between neurons involved in border or contour detection very important in figure-ground segregation processes.

Because we achieve invisibility through contrast decrease, it is important to identify whether the neural responses we report resemble those reflecting directly the physical change or whether they could rather reflect the subjective status of stimulus perception. In our study physical contrast decreases implies local luminance increases of the stimulus. Luminance variations and contrast adaptation are known to modulate signal processing in PVC in animals and humans (Movshon and Lennie, 1979; Carandini and Ferster, 1997; Gardner et al., 2005; Peng and Van Essen, 2005; Geisler et al., 2007; Xing et al., 2014). But these effects on occipito-temporal cortices are yet poorly understood. Single neurons and neuronal populations in these regions display less sensibility to low-level stimulus features and respond robustly to high-level integrated objects such as stimulus semantic categories (Desimone et al., 1984; Kreiman et al., 2000; Quiroga et al., 2005; Vidal et al., 2010) and conscious perception (Quiroga et al., 2008b).

### BROADBAND GAMMA ACTIVITY MODULATION DURING PERCEPTUAL SUPPRESSION IN HUMAN CORTEX

Previous electrophysiological measures from monkey PVC showed that luminance decreases produce an increase in neuronal spiking and gamma-band power as compared to luminance increases which tend to suppress neuronal population discharges (Xing et al., 2014). Xing et al. (2014) concluded on the existence of different cortical mechanisms behind brightness adaptation and argued that changes in neuronal firing in V1 neurons following brightness/darkness adaptation modulate the balance between local excitatory and inhibitory networks, thereby affecting global population discharge rates (Peng and Van Essen, 2005; Xing et al., 2014). The broadband gamma amplitude increase observed in our study following stimulus contrast decrease elicited an opposite effect, observed throughout various occipital, temporal and fronto-insular recording sites, but surprisingly not in PVC. Though we currently have no explanation for the differences in PVC between Xing et al. (2014) study and ours, we suggest that it may involve neuronal adaptation effects caused by the prolonged exposure to the same stimulus. These effects probably originate at retinal level (Burbeck and Kelly, 1984; Hunzelmann and Spillmann, 1984).

Perceptual suppression induced with general flash suppression paradigm has been shown to negatively modulate broadband gamma activity in macaque V4, especially when also negatively modulating multi-unit activity during stimulus offset (Wilke et al., 2006). However, such effects were absent in V1 (Wilke et al., 2006; Maier et al., 2008; Keliris et al., 2010) and in certain nuclei of the thalamus (Wilke et al., 2009). Here we report that the positive modulation of gamma-band activity during stimulus invisibility occurs on a majority of responding sites located in occipital and temporal cortex. These responses correlated with PERCEPTUAL and SUPPRESSION effects and suggest the existence of local networks involved in the active processing of the subjective appearance and disappearance of a visual stimulus. On a related note, neuronal contrast polarity OFF (and ON) signals are processed early on within the visual pathway from the retina to V1 including the thalamus (Hartline, 1938; Kuﬄer, 1953; Tanaka, 1983; Reid and Alonso, 1995; Jin et al., 2008, 2011; Yeh et al., 2009). A similar type of neural response could exist to signal perceptual disappearances in higher levels of the cortical hierarchy. Noteworthy, this observation takes place in an experimental setting which does not involve a competing interaction with a newly presented stimulus or information stream, thereby discarding any relation to novel stimulus representation processing which might involve spatial, feature, or object selective visual attention, and usually increase gamma-band activity (Fries et al., 2001) but see Chalk et al. (2010) for opposite effects of attention on V1 neural activity. Yet, as mentioned before, our results remain conditioned by possible sensory adaptation effects which are inherent to the PS paradigm that was used. Future studies will need to clarify this relationship.

Finally, though we did not record eye-movements during this study, we suggest that the reported effects, especially in the PS condition (INV), do a priori not reflect saccadic effects. A recent intracranial study showed that peri-saccadic gamma-band activity is positively modulated in medial regions of PVC (Uematsu et al., 2013) without any further effects in all extra-occipital regions. Yet, in our study, we found condition specific effects throughout different cortical regions, and recordings sites in PVC elicited either no gamma-band response to negative contrast changes (INV), or delayed responses to positive contrast changes (VIS) beyond the reported saccade-induced latency effects.

### GLOBAL LOW-FREQUENCY AMPLITUDE MODULATIONS BY STIMULUS INVISIBILITY

Changes in low-frequency oscillations have been associated with state changes of cortical networks and cortical communication (Siegel et al., 2012); sustained power suppressions in sensory regions often relate with active local neural processing, sensory change processing but also modulation by cognitive processes such as attention and consciousness (Fries et al., 2001; Gaillard et al., 2009; Levy et al., 2013). Previous studies reported a sustained low-frequency activity decrease of the LFP in early visual cortex, V1–V4, following target invisibility by general flash suppression (Wilke et al., 2006; Maier et al., 2008). We report this same type of effect generalized across extra-striate visual cortices in the occipito-temporal region, such as lateral occipital cortex, temporal cortex (including fusiform gyrus, middle temporal gyrus) but also frontal and insular cortices. The extent of this finding across participants and within this wide variety of cortical areas beyond PVC stresses the generality of this effect and the cortically distributed processing involved in PS and stimulus invisibility. However, while low-frequency amplitude decreases occurred massively, they did not systematically coincide with simultaneous broadband gamma activity increases. This is similar to previous observations from monkey brain area V1 where either neuronal discharge rates or gamma-band power were modulated independently from alpha-band oscillatory power (Wilke et al., 2006; Maier et al., 2008). In fact, in these studies either neuronal discharges or broadband gamma activity did not correlate to stimulus visibility status, thereby suggesting that very early sensory neural structures might not directly enable or be the initiator of conscious perception processing. Low-frequency power suppression across a large proportion of cortices, sensory and non-sensory, could thus indicate a profound change of state as has been suggested previously for beta-band oscillations (Engel and Fries, 2010; Panagiotaropoulos et al., 2013; Rey et al., 2014) hence not being directly involved in the processing of the content of conscious perception.

### STIMULUS VISIBILITY-RELATED NEURAL MODULATIONS IN PRIMARY VISUAL CORTEX

The neural emergence analysis of the gamma-band activity in PVC reveals three important pieces of information: (1) stimulus offset (OFF) induces an immediate positive response, terminating the adaptation period, (2) contrast decrease (INV) elicits no response, and (3) a late positive amplitude increase occurs for stimulus contrast increase, around 300 ms, while no early reaction similar to the OFF condition is observed. It appears as if the early progressive negative amplitude modulation in VIS (<300 ms) does not directly reflect the contrast increase.

The absence of gamma-band activity modulation during PS in V1 may be caused by a silencing of neuronal activity caused by forced adaptation of downstream retinal cell populations. If a contrast decrease reduces the sensory gain of edge neurons in these populations, the figure-ground segregation process might be compromised and produce a relative gain increase for background neurons, relayed upstream to V1. This could bias the competitive interactions and entail a filling-in phenomena. However, as suggested by previous studies (Wilke et al., 2006; Maier et al., 2008; Keliris et al., 2010) and current discussions on the role of V1 in conscious visual perception, it is possible that this structure though necessary may not be sufficient for visual awareness, and that this function rather concerns frontal and temporal cortices which could consolidate a cortico-thalamo-cortico network that conveys the content of conscious experience (Leopold, 2012; Panagiotaropoulos et al., 2014).

The neural emergence pattern of the VIS condition hints towards a local network activation around 300 ms. This event could reflect local cortical activation triggered by distant upstream networks. This interpretation is in line with theoretical concerns on the necessary involvement of frontal feedback signals in the ignition of conscious processing in cortical networks (Dehaene and Changeux, 2011; Boly et al., 2013). But if this delayed gamma-band rebound reflects top–down processing, why did it not result in a PERCEPTUAL effect? On the one hand we can be critical about our contrasts effectiveness. On the other hand, this effect could constitute the first electrophysiological example of a salience-driven attention effect without the underlying conscious processing, as suggested by previous neuroimaging data (Watanabe et al., 2011) and as discussed by others (Leopold, 2012; Panagiotaropoulos et al., 2014). This interpretation, however, needs further investigation.

A previous human intracranial study using visual masking with string stimuli observed a general increase of broadband gamma activity across all recording sites between 200 and 300 ms (Gaillard et al., 2009). However, the authors did not single out, or did not have, activity from PVC, though they showed average lobe-specific activity (ex: occipital lobe). They reported earlier visual masking effects in the gamma-band in the occipital lobe than in the frontal lobe. In our study, PERCEPTUAL effects were observed first in fronto-insular networks and then in temporal and occipital visual areas, but not PVC. Another human intracranial study also using visual masking but contrasting consciousness based on perceptual reports exclusively, focused on single recording sites within the occipito-temporal cortex (Fisch et al., 2009). In this study, the broadband gamma activity showed a high degree of category selectivity for complex visual stimuli such as faces, houses, objects, etc., and similar to the study of Gaillard et al. (2009) also reported differential effects related to conscious perception between 250 and 300 ms. However, there was no significant effect of conscious stimulus recognition in the broadband gamma range within low-level visual areas. Considering the lack of evidence of similar effects in V1 of the macaque brain during PS (Wilke et al., 2006), our results in the broadband gamma range suggest that PVC is not primarily involved in time in the emergence of visual consciousness. However, the sudden positive amplitude rebound suggest that a modulatory influence related to stimulus visibility, maybe from the involvement of top–down processes, reaches PVC around 300 ms. The choice of the three conditions in our contrastive analysis may also not be optimally matched so as to reveal conscious perception effects (i.e., PERCEPRTUAL). Importantly, the possibility of a significant contribution of adaptation effects to our result pattern remains to be discarded in future studies.

Low-frequency PERCEPTUAL effects started at 350 ms and were coincident with significant amplitude increases in the VIS condition and amplitude decreases in the INV condition. From a more global perspective, this result is coherent with low-frequency responses observed during PS in macaque V1 (Wilke et al., 2006) but also within subcortical structures such as in the pulvinar (Wilke et al., 2009). However, recordings in the latter only showed modulations of low-frequency power when the animal was actively involved in reporting its perception, and not when passively viewing the perceptual alternations. In our study this power modulation was not dependent on behavioral report. Moreover, our results are incongruent with the effects in the low-frequency band (i.e., beta band) reported by Gaillard et al. (2009) in human occipital lobe in both modulation latency and sign, which occurs earlier and is positive. As stated previously in the introduction, this difference may be due to specifics of physical stimulation, stimulus adaptation, and behavioral task within experimental designs used in these studies: PS and masking use within their contrastive analyzes qualitatively different types of invisibilities which probably also elicit different neural responses. In masking paradigms, stimulus invisibility is induced on the path toward conscious perception, however, never reaching it. In PS, stimulus invisibility occurs after the stimulus has reached a conscious status.

### INSULAR AND FRONTAL CONTRIBUTIONS TO PERCEPTUAL SUPPRESSION AND CONSCIOUS PERCEPTION

Most broadband gamma activity modulation by PERCEPTUAL effects located in occipito-temporal cortex (excluding PVC). Yet, we also found these responses in anterior insular cortex and inferior frontal cortex with overall onset latencies much earlier than those reported in visual areas. In the midst of the ongoing discussions on the respective participation of V1 and frontal regions in conscious perception (Leopold, 2012; Safavi et al., 2014) our results are coherent with current experimental results showing modulation of neuronal activity in frontal networks correlated to stimulus visibility (Libedinsky and Livingstone, 2011; Panagiotaropoulos et al., 2012). Because (1) it has been recently shown that AI is structurally connected to posterior visual areas including temporal cortex (Jakab et al., 2012), (2) that AI has been shown to concentrate Von Economo neurons (Nimchinsky et al., 1999) which could transmit at very high speed information through long-distance connections in the brain, and (3) based on the latencies of our broadband gamma PERCEPTUAL effects, we suggest that in our PS paradigm conscious perception’s neural signaling emerges primarily in AI and may then propagate to IFG and other high-level cortical structures to generate top–down influences upon the downstream sensory networks. AI could also directly influence visual processing based on its direct connections with these networks (Jakab et al., 2012). This observation and proposal will need to be verified in future studies. The later emerging PERCEPTUAL effects in the low-frequency band in AI may thus be signaling a consequence of the first (and probably feed the forward sweep) of consciouss perception.

Why do insular networks produce increased broadband gamma activity for stimulus disappearance? First, anterior insula networks have been associated with the processing of perceptual saliency (Seeley et al., 2007; Corbetta et al., 2008) and the effects we report may reflect the high saliency value for sudden stimulus disappearance. This does, however, not explain the opposite pattern, broadband gamma decrease, for stimulus re-appearance (VIS). Moreover, neural activity decrease in this region is temporally related to upcoming lapses of attention and mind wandering (Weissman et al., 2006; Mason et al., 2007) thereby negatively affecting conscious sensory processing, while in our study it is related to a gain in conscious perception through stimulus visibility. Current models on the role of the insula suggest it performs a complex integration of interoceptive and perceptual information thereby possibly generating a unified conscious feeling of self (Craig, 2009). It is therefore unlikely that the effects reported in this brain region are related to low-level feature coding like the ones related to contrast coding in PVC. The fronto-insular perceptual effects we report could thus reflect the first emergence of conscious visual perception from the top of the cortical hierarchy (Hochstein and Ahissar, 2002).

## Conflict of Interest Statement

The authors declare that the research was conducted in the absence of any commercial or financial relationships that could be construed as a potential conflict of interest.
